# Ultrahigh Pressure Processing Produces Alterations in the Metabolite Profiles of *Panax ginseng*

**DOI:** 10.3390/molecules21060816

**Published:** 2016-06-22

**Authors:** Mee Youn Lee, Digar Singh, Sung Han Kim, Sang Jun Lee, Choong Hwan Lee

**Affiliations:** 1 Department of Bioscience and Biotechnology, Konkuk University, Seoul 05029, Korea; kkamlice@hanmail.net (M.Y.L.); singhdigar@gmail.com (D.S.); 2 Nutrex Technology Co., Seongnam, Gyeonggi-do 13494, Korea; ksh@nutrex.co.kr; 3 Holistic Bio Co., Seongnam, Gyeonggi-do 13494, Korea

**Keywords:** *Panax ginseng*, ultrahigh pressure, antioxidant activity, mass spectrometry, multivariate analyses

## Abstract

Ultrahigh pressure (UHP) treatments are non-thermal processing methods that have customarily been employed to enhance the quality and productivity of plant consumables. We aimed to evaluate the effects of UHP treatments on ginseng samples (white ginseng: WG; UHP-treated WG: UWG; red ginseng: RG; UHP-treated RG: URG; ginseng berries: GB; and UHP-treated GB: UGB) using metabolite profiling based on ultrahigh performance liquid chromatography-linear trap quadrupole-ion trap-tandem mass spectrometry (UHPLC-LTQ-IT-MS/MS) and gas chromatography time-of-flight mass spectrometry (GC-TOF-MS). Multivariate data analyses revealed a clear demarcation among the GB and UGB samples, and the phenotypic evaluations correlated the highest antioxidant activities and the total phenolic and flavonoid compositions with the UGB samples. Overall, eight amino acids, seven organic acids, seven sugars and sugar derivatives, two fatty acids, three notoginsenosides, three malonylginsenosides, and three ginsenosides, were identified as significantly discriminant metabolites between the GB and UGB samples, with relatively higher proportions in the latter. Ideally, these metabolites can be used as quality biomarkers for the assessment of ginseng products and our results indicate that UHP treatment likely led to an elevation in the proportions of total extractable metabolites in ginseng samples.

## 1. Introduction

Ginseng, a renowned traditional herbal remedy, is commonly associated with the dried roots of several species in the genus *Panax* of the family Araliaceae [1]. Major commercial ginseng products are derived from *P. ginseng* (Korean ginseng), *P. quinquefolius* (American ginseng), and *P. notoginseng* (notoginseng) [2]. Of these, *P. ginseng* is endemic to Korea, Japan, and China, and it has been cultivated in these countries for more than 4000 years [3]. Its various commercial analogs include fresh ginseng, red ginseng, white ginseng, and sun ginseng. Ginseng root supplements are reported to improve diabetic conditions, immune functions, cardiovascular conditions, psychological functions, prevent certain cancers, and reduce fatigue [4,5,6]. The major active components of ginseng are the ginsenosides, a diverse group of steroidal saponins. These components are differentially distributed in different plant parts, including the roots, leaves, seeds, and berries. Therefore, each part of the ginseng plant exhibits distinct ginsenoside profiles with a myriad of pharmacological effects [7].

Ginseng berries, the nearly drupaceous fruit of *P. ginseng,* are generally more efficacious than ginseng seeds and roots in terms of total ginsenoside contents. The fruits turn red upon ripening in autumn and produce harvestable seeds following the third year of growth. Ko *et al.* [8] reported significantly higher levels of total ginsenosides and ginsenoside from ginseng berries than from four-year-old cultivated ginseng seeds and roots. Furthermore, recent studies using mouse models demonstrated that ginseng berry extracts exhibited potentially stronger antihyperglycemic and anti-obesity effects than those of its root extracts [9,10].

Ultrahigh pressure (UHP) processing is a modern non-thermal food preservation technology under which a product is subjected to a high-pressure (50–1000 MPa) in order to increase its shelf life [11]. UHP or HHP treatments are carried out at room temperature without any heat processing under isostatic and adiabatic conditions, with the exception of a marginal (<3 °C) temperature rise due to compression [12]. Moreover, UHP processing is more economical than analogous thermal treatments, and it markedly increases the rate at which deleterious microbes and enzymes are inactivated [13]. UHP processing methods have conventionally been applied to milk, fruit, and vegetable products [14]. In addition to maintaining freshness, the method has been used to enhance the safety, productivity, quality and extraction yields of food products [15]. Moreover, a recent surge in the number of scientific reports on the topic also implies increased usage of UHP methods in food and nutrition research. Regarding ginseng extraction, UHP processing reportedly influences the levels of crude saponins and total ginsenosides [16].

The metabolomics has emerged as a cognitive tool to explore the impact of different processing methods on the nutritional quality and desired flavors of food products [17]. Recent advancements in analytical instrumentation, including nuclear magnetic resonance (NMR), capillary electrophoresis (CE), and liquid chromatography (LC) or gas chromatography (GC) coupled to mass spectrometry (MS), have led to a leap in food and plant metabolomics because of the possibility of performing high-throughput processing [18,19,20]. The growing impetus towards the application of metabolomics in a variety of scientific fields is evident from the number of significant reports on different plants, microorganisms, and environmental systems [21,22,23]. The LC-MS and GC-MS analytical platforms are usually been employed to gain an insight into plant metabolomes following subjection of the test samples to an experimental condition. Previously, the studies involving the UHP-processed ginseng metabolite profiling indicated their differential saponin contents and associated efficacies [24].

In this investigation, we highlighted the effects of UHP processing on Korean ginseng metabolomes using MS-based metabolite profiles coupled with multivariate analysis. We also investigated the associated bioactivities (using e.g., 2,2′-azinobis-3-ethylbenzotiazoline-6-sulfonic acid diammonium salt (ABTS) and ferric reducing antioxidant power (FRAP) method, total phenolic content (TPC), and total flavonoid content (TFC) assays) of different ginseng samples and correlated the phenotypes with the corresponding levels of specific metabolites. Hence, the present study comparatively evaluates different commercial ginseng types and the effects of the UHP processing method on their potential efficacies and phenotypes.

## 2. Results

### 2.1. Comparative Evaluation of Different Ginseng Samples Based on Metabolite Profiles and Antioxidant Activities

**Figure 1:**
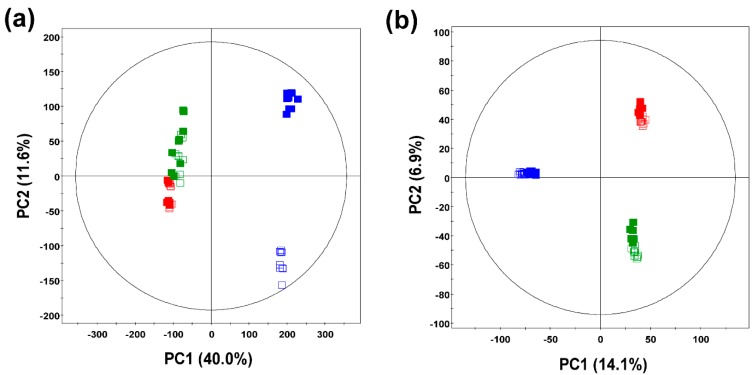
PCA score plots of *P. ginseng* samples analyzed by (**a**) gas chromatography time-of-flight mass spectrometry (GC-TOF-MS) and (**b**) ultrahigh performance liquid chromatography-linear trap quadrupole-ion trap-tandem mass spectrometry (UHPLC-LTQ-IT-MS/MS). □: non-treated white ginseng (WG); ■: UHP-treated white ginseng (UWG), □: non-treated red ginseng (RG); ■: UHP-treated red ginseng (URG), □: non-treated ginseng berry (GB); ■: UHP-treated ginseng berry (UGB).

**Figure 2:**
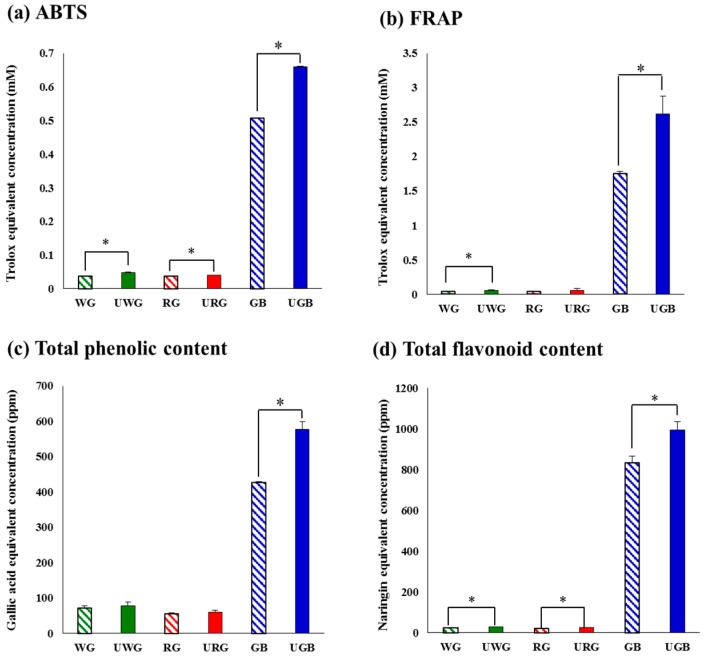
Antioxidant activity assay (**a**) diammonium salt (ABTS); (**b**) fluorescence recovery after photobleaching (FRAP); (**c**) total phenolic contents; and (**d**) total flavonoid contents of *Panax ginseng* samples. Each value is expressed as mean ± SD (* *p* < 0.05, paired sample *t-*test). ▧: non-treated white ginseng (WG), ■: UHP-treated white ginseng (UWG), ▧: non-treated red ginseng (RG), ■: UHP-treated red ginseng (URG), ▧: non-treated ginseng berry (GB), ■: UHP-treated ginseng berry (UGB).

The multivariate principal component analysis (PCA) method was employed to distinguish the six groups of commercial ginseng samples and to interpret their intrinsic similarities based on their chromatographic profiles. As shown in Figure 1a, the PCA plot for the gas chromatography time-of-flight mass spectrometry (GC-TOF-MS) data indicated that the metabolite profiles for ginseng berry products (GB and UGB) and other ginseng products (WG, UWG, RG, and URG) were clearly separated by PC1, whereas the ginseng berry products were separated along PC2. The PCA score plot based on GC-MS data explained 51.6% of the total variability (PC1: 40.0%; PC2: 11.6%). In contrast, the PCA score plot for the ultrahigh performance liquid chromatography-linear trap quadrupole-ion trap-tandem mass spectrometry (UHPLC-LTQ-IT-MS/MS) data showed that the ginseng berry products were clearly separated from other ginseng products along PC1 (14.1%), whereas the white ginseng products (WG and UWG) and the other ginseng products (GB, UGB, RG, and URG) were discriminated by PC2 (6.9%) (Figure 1b). The associated bioactivities for different experimental ginseng groups were measured by focusing on the antioxidant activities (ABTS and FRAP), TPC, and TFC (Figure 2).

The observed antioxidant activity for ginseng berry products was significantly higher than the other ginseng products. Furthermore, the UHP-treated groups exhibited marginally higher antioxidant activities than the non-UHP-treated groups. Similarly, the TPC and TFC assays also indicated significantly higher levels of phenolic and flavonoid metabolites among the different ginseng berry groups (GB and UGB). The observed bioactivities and phenotypic levels were consistently higher for UHP-treated ginseng berry products compared to the non-treated ones (*p* < 0.05).

### 2.2. Identification of Significantly Discriminant Metabolites in Ginseng Berry Products Based on UHP Treatment

**Figure 3:**
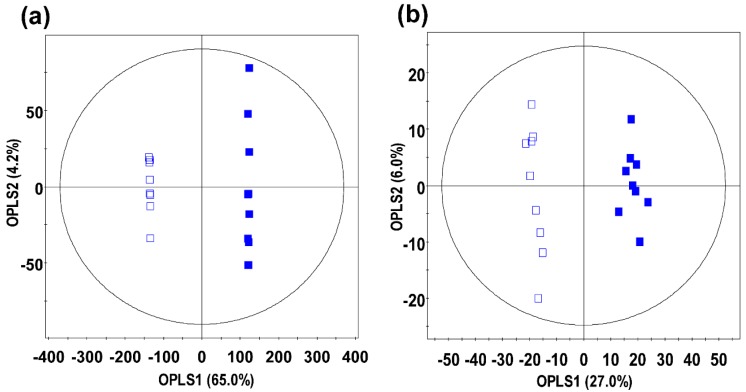
Orthogonal projection to latent structures-discriminant analysis (OPLS-DA) score plots analyzed using the (**a**) GC-TOF-MS and (**b**) UHPLC-LTQ-IT-MS metabolite datasets for ginseng berries. □: non-treated ginseng berry (GB), ■: UHP-treated ginseng berry (UGB).

Among the different groups of ginseng samples analyzed, the ginseng berry products (GB and UGB) were clearly distinguished based on their distinct metabolite profiles and significantly higher phenotypic levels (*i.e.*, antioxidant activity, phenolic contents, and flavonoid contents). The primary and secondary metabolite variations for UHP-treated ginseng were analyzed using GC-TOF-MS and UHPLC–LTQ-IT-MS methods, respectively. The representative GC-MS and LC-MS chromatograms for both the samples are provided as Supplementary Materials (Figure S1). Subsequently, the metabolite profiles were statistically analyzed using the orthogonal projection to latent structures-discriminant analysis (OPLS-DA) model to determine the metabolite variants among the non-treated and UHP-treated ginseng berry products (Figure 3). Significantly different metabolites were selected from OPLS-DA models, based on the variable importance projection (VIP) analysis results (>0.7) at *p*-values (<0.05), and the identified metabolites were annotated based on their retention time and standard mass fragmentation patterns.

The selected metabolites from statistical analyses of the GC-TOF-MS and UHPLC-Q-TOF-IT-MS datasets are shown in Table 1 and Table 2, respectively. Further, the molecular formulae of the metabolites were determined using GC-TOF-MS data analysis, followed by a National Institute of Standards and Technology (NIST) library search and annotation. The GC-TOF-MS analysis enabled the identification of 24 significantly discriminant primary metabolites viz., serine, threonine, aspartic acid, γ-aminobutyric acid (GABA), hydroxyglutaric acid, glutamic acid, asparagine, tryptophan, lactic acid, ethylmalonic acid, malic acid, shikimic acid, citric acid, gluconic acid, glyceric acid, *meso*-erythritol, ribitol, galactose, glucose, *myo*-inositol, turanose, linoleic acid, and stearic acid in the UHP-treated ginseng berries (Table 1). In addition, 13 secondary metabolites viz., notoginsenoside R1, ginsenoside-Re, ma-ginsenoside Re, ginsenoside Rb1, notoginsenoside R2, ginsenoside Rd, malonyl-ginsenoside Rd, notoginsenoside Rt1, malonyl-notoginsenoside Rt1, ginsenoside Rg3, and three unidentified metabolites were found significantly discriminant for the ginseng berry groups using the UHPLC-LTQ-IT-MS data (Table 2).

The selected sets of metabolites were tentatively identified based on their respective molecular weights, elemental compositions, and mass errors (mDa) using MassLynx v4.1 analytical software. Additionally, the MS^n^ fragmentation and UV λmax (nm) data from UHPLC-LTQ-IT-MS/MS analyses were used to support the metabolite identification information.

### 2.3. Relative Contents of Metabolites among the Non-treated (GB) and UHP-Treated (UGB) Ginseng Berry Samples and their Visual Representation in Biosynthetic Pathways

**Figure 4:**
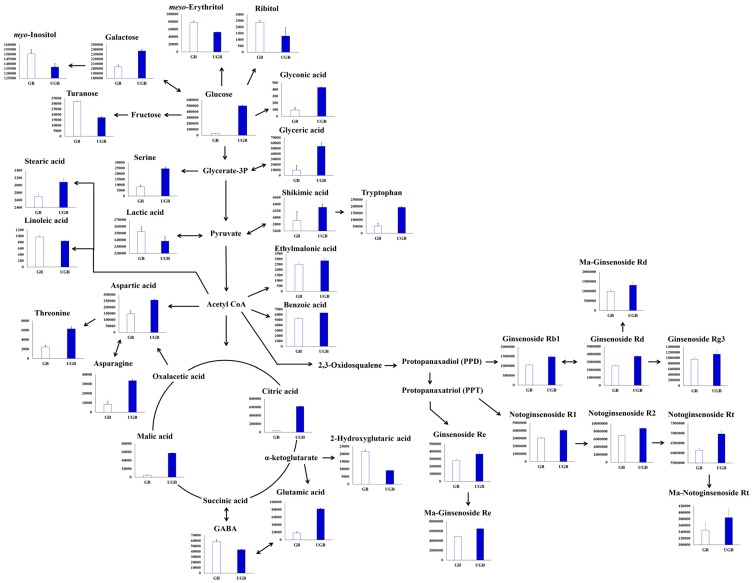
Schematic representation of the relative metabolite contents visualized in respective metabolic pathways for ginseng berry samples (GB and UGB) that correspond to UHP treatments. The pathways were modified from the Kyoto Encyclopedia of Genes and Genomes (KEGG) database (http://www.genome.jp/keg/). The Y-axis of the histogram represents peak areas for respective metabolites. Data are mean values, and the error bars represent standard deviation values (*n* = 9). These metabolites were selected by VIP > 0.7 and *p* < 0.05.

As shown in Figure 4, the relative changes in the metabolite levels were estimated and visualized in the respective metabolic pathways, which corresponded to the UHP processing for ginseng berry samples. The relative metabolite levels were calculated using the corresponding peak area in the chromatograms. The average values for the detected metabolites from the LC and GC chromatograms are shown in Supplementary Table S1. For the metabolic pathway visualization, the relative contents of many metabolites varied among the GB and UGB samples.

In particular, the levels of serine, threonine, aspartic acid, hydroxyglutaric acid, glutamic acid, asparagine, and tryptophan were apparently increased by UHP-treatment, whereas that of GABA was decreased. With exception to lactic acid, the relative levels of organic acids (e.g., ethylmalonic acid, malic acid, benzoic acid, shikimic acid, citric acid, and gluconic acid) were elevated in UGB samples. On the other hand, the relative proportions of seven discriminant sugars and sugar derivatives in the GB and UGB samples were markedly different. In particular, monosaccharides (galactose and glucose) and sugar acid (glyceric acid) were increased in UGB samples. However, the sugar alcohols (*meso*-erythritol, ribitol, and *myo*-inositol) and disaccharides (turanose) were contrastingly derogated in UGB samples. Stochastically, the putative levels of fatty acids in the UGB samples also varied when the relative contents of linoleic acid and stearic acid were decreased and increased, respectively. Moreover, the relative proportions of 10 ginsenosides were increased in UHP-treated samples compared to non-treated ginseng berries. Therefore, our results indicated that UHP treatment presumably led to specific elevations in the levels of extractable primary and secondary metabolite contents in ginseng berry samples.

## 3. Discussion

UHP treatment has emerged as an attractive non-thermal food processing technology with great potential for the amelioration of shelf life and organoleptic properties of stored food products. In particular, UHP treatments are reported to initiate an increase in the relative contents of crude saponin and ginsenoside in processed ginseng products [28], enhancing their pharmacological and nutritional values [29,30]. These prophylactic metabolites are distributed throughout the ginseng plant (*i.e.*, root, leaf, flower, and berry), which is evident from ginsenoside profiling studies [31].

In this study, we elucidated the effects of UHP treatments on the composite metabolomes for the three classes of ginseng products (white ginseng, red ginseng, and ginseng berries). The untargeted metabolite profiles for the non-treated (WG, RG, and GB) and UHP-treated (UWG, URG, and UGB) ginseng products were examined using the GC-TOF-MS and UHPLC-LTQ-IT-MS/MS methods with multivariate analyses.

As shown in Figure 1, we sought to separate each ginseng sample based on primary and secondary metabolite profiles using PCA. Ginseng berry samples were critically distinct from the rest of the ginseng products based on substantial distinction between the GB and UGB in the PCA score plots based on GC-TOF-MS datasets (Figure 1a). Regarding the phenotypes, UHP-treated ginseng samples consistently exhibited higher levels of antioxidant activities as well as phenolic and flavonoid compounds compared to the non-treated (GB) samples (Figure 2). Astonishingly, the phenotypic levels were several folds higher in GB and UGB samples compared to the other ginseng types, which implies their higher pharmacological potential with free radical oxygen scavenging and anticancer activities [32].

As shown in Figure 3, the OPLS-DA model clearly demarcated the discriminant metabolites between GB and UGB samples and maximized the statistical separation with satisfactory goodness of fit values for both the GC-TOF-MS (R^2^X = 0.607, R^2^Y = 0.997, and Q^2^ = 0.996) and UHPLC-LTQ-IT-MS/MS (R^2^X = 0.270, R^2^Y = 0.988, and Q^2^ = 0.946) datasets, suggesting their high predictive accuracies. In Table 1 and Table 2, primary and secondary metabolites were selected as differential variables using VIP values (VIP > 0.7). Twenty-four primary metabolites, including eight amino acids, seven organic acids, seven sugars and sugar derivatives, and two fatty acids were significantly different between GB and UGB, based on the GC-TOF-MS dataset analysis (Table 1). Similarly, the secondary metabolite profiles were examined, and five ginsenosides, three malonyl-ginsenosides, and three unknown metabolites differed significantly between the GB and UGB groups (Table 2).

In addition to ginsenosides (ginseng saponins), the ginseng plant is reported to contain a spectrum of functional metabolites (e.g., phenolic compounds, polysaccharides, peptides, polyacetylenic alcohols, fatty acids, amino acids, and organic germanium) with a number of pharmacological effects [33]. UHP processing is reported to modulate the properties of food components by altering the phenolic acid contents, proanthocyanidin structure, and protein stabilization [34,35,36]. In agreement with previous reports, we also observed significant elevations in the relative levels of the various primary and secondary metabolites in the UHP-treated samples, and illustrated using a metabolic pathway representation (Figure 4). Specifically, amino acids play an important role in the defense and stress response mechanisms in plants, despite being pivotal in food quality and safety with regard to human consumption [37,38].

In this study, we found that seven amino acids (serine, threonine, aspartic acid, hydroxyglutaric acid, glutamic acid, asparagine, and tryptophan) were highly accumulated in UGB, and these amino acids decisively contribute to the nutritional quality of ginseng. Notably, aspartic acid helps to enhance muscle building, sperm count, and energy metabolism, and asparagine is known to improve male fertility through enhanced sperm production and motility, despite being essential for desired sperm morphology. Similarly, tryptophan, the precursor of the “happiness hormone” serotonin, indirectly affects the amount of serotonin in brain, and serotonin levels in the brain regulate vital neural functions (e.g., mood, sleep, appetite, memory, and learning) [39]. With the exception of GABA, the relative levels of organic acids (e.g., ethylmalonic acid, malic acid, benzoic acid, shikimic acid, citric acid, and gluconic acid) were higher in UGB groups. Functionally, malic acid and citric acid are well known acidulants, which influence the palatability of food through pH adjustments [40]. The necessary titers of organic acids are often considered crucial in terms of the desired flavors and textures during food processing [41]. Benzoic acid and shikimic acid naturally exist in many plants and serve as the important precursors for the biosynthesis of many other organic substances [41,42]. Further, benzoic acid is usually known to relieve skin ailments such as acne, ringworm, and tinea pedis [43], whereas shikimic acid is often associated with pharmacological effects such as anticancer, antioxidant, and antibacterial properties [44,45].

Recently, *Panax* species were reported to contain a variety of bioactive ingredients, including ginsenosides, fatty acids, and polysaccharides with a myriad of health benefits (e.g., immuno-modulatory, antibacterial, anti-oxidative, antidepressant, anti-septicemic, and anti-inflammatory activities) [46]. Our results showed that the contents of monosaccharides (galactose and glucose) and sugar acid (glyceric acid) were comparatively higher in UGB groups. Physiologically, the higher levels of sugar and sugar derivatives primarily influence glycolysis and the citric acid cycle, which provide the material and energy necessary for the synthesis of complex biomolecules, including amino acids [47]. Therefore, the content of sugars and their metabolic pathways are important for amino acid synthesis. We detected higher levels of galactose, glucose, and *myo*-inositol in UGB groups, which were previously reported as potential biomarkers for ginseng quality assessment [48]. Among the fatty acids, only linoleic acid and stearic acid were detected as significantly discriminant metabolites between GB and UGB groups. We showed that, in contrast to the elevated levels of stearic acid, linoleic acid contents were decreased in UGB groups. As a functional nutrient, stearic acid is known to inhibit the absorption of cholesterol in obese mouse models [49].

The ginsenosides found almost exclusively in *Panax* (ginseng) has a long history of usage in traditional oriental medicines [7]. Several functional properties are associated with ginsenosides (e.g., anti-allergic, anti-carcinogenic, anti-inflammatory, and immunomodulatory effects) [50,51]. These contents also vary significantly with biological and environmental factors. Our study further indicated notoginsenosides (R1, R2, and Rt1), ginsenosides (Re, Rd, and Rg3), and malonyl-ginsenosides (R1, R3, and Rt1) as significantly discriminant metabolites among GB and UGB groups, with latter containing their comparatively higher levels. UHP presumably affects the inter- and intra-molecular chemical bonds in proteins. Moreover, the high pressure likely breaks weak interactions such as hydrogen, hydrophobic, and electrostatic bonds in biological membranes, but it does not affect the covalent bonds [16]. Hence, the method enhances the shelf life of food without compromising its nutritional, functional, and organoleptic aspects [52]. Therefore, the higher extraction yield of ginsenosides in UHP-treated ginseng samples might result from pressure-mediated bond weakening and breakage [53].

Overall, our study suggested that UHP treatment somehow causes an elevation in the nutrient components of ginseng berries. Here, our results are in agreement with earlier reports, which suggested that high-pressure treatment of commodities like onion, apple [54], pear [55], and ginseng [56] results in elevated phenolic components that enhance their antioxidant activity. These reports suggested that UHP treatment alters membrane permeability, causing disruptions in cell walls and releasing aromatic and phenolic compounds from tissues, which in turn improves their extractability [57]. This work further emphasized the importance of MS-based metabolite profiling coupled with multivariate analyses as an effective cognitive tool to discern the quality and safety of processed foods.

## 4. Experimental Section

### 4.1. Chemicals and Reagents

Acetonitrile, methanol, water, and hexane were purchased from Fisher Scientific (Pittsburgh, PA, USA). Methoxyamine hydrochloride, *N*-methyl-*N*-(trimethylsilyl) trifluoroacetamide (MSTFA), pyridine, formic acid, 6-hydroxy-2,5,7,8-tetramethylchroman-2-carboxylic acid (Trolox), gallic acid, naringin, hydrochloric acid, potassium persulfate, 2,2′-azinobis (3-ethylbenzothiazoline-6-sulfonic acid) diammonium salt (ABTS), acetic acid, sodium acetate, iron (III) chloride hexahydrate, 2,4,6-tris(2-pyridyl)-s-triazine (TPTZ), hydrochloride, diethylene glycol, sodium hydroxide, Folin-Ciocalteu’s phenol reagent, sodium carbonate, and standard compounds were all purchased from Sigma–Aldrich (St. Louis, MO, USA). All chemicals and solvents used were of analytical grade.

### 4.2. Preparation of Ginseng Materials

Freshly harvested four-year-old Korean ginseng roots (white ginseng (WG), *P. ginseng* C.A. Meyer) and berries (ginseng berry, GB) that were cultivated in Geumsan Country (Chungcheong Province, Korea) were used. Raw WG samples (20 kg each) were washed with tap water to remove impurities from the surface. Raw samples of GB, which consisted of the berry skin and pulp without the seeds (10 kg each), were prepared and vacuum packaged in polyethylene film (thickness = 80 µm). Both the WG and GB samples were twice subjected to UHP treatment at 550 MPa for 1 min each using UHP equipment (Quintus Food Press QFP 35L-600, Avure Technologies AB, Vasteras, Sweden), and water was used to generate the high pressure. Samples (10 kg) of WG and UHP-treated WG (UWG) were steamed (95 °C for 3 h) and dried (53 °C for 70 h) to make RG and URG samples, respectively. All of the non-UHP-treated and UHP-treated samples were finely pulverized using a mortar and pestle, and the samples were then stored at −20 °C until analyses were conducted.

### 4.3. Sample Preparation for Chromatographic Analyses

Ginseng samples for GC-TOF-MS and UHPLC-LTQ-IT-MS/MS analyses were prepared using the modified protocols of Welthagen [58] and Li [59], respectively. Twenty mg of the dried sample powder was dissolved in 1 mL of 70% methanol through vortexing for 10 s. The samples were then sonicated in an ultrasonic water bath (Power Sonic 305, Hwashin Technology Co., Seoul, Korea) for 1 h, and were subsequently centrifuged at 12,000 rpm for 10 min at 4 °C. The resulting supernatant was filtered using a 0.2-μm PTFE filter and transferred to Eppendorf tubes. The extract solution was subsequently evaporated using a speed-vacuum apparatus (Biotron, Seoul, Korea). For the GC-TOF-MS analysis, an additional two-stage chemical derivatization procedure was performed. First, oximation was conducted by dissolving the dried extracts in 50 μL of methoxyamine hydrochloride (20 mg/mL in pyridine for 90 min at 30 °C), followed by silylation using 50 μL of MSTFA (30 min at 37 °C). For UHPLC-LTQ-IT-MS/MS analysis, dried extracts were re-dissolved in methanol and syringe-filtered (0.2 μm PTFE filter, Chromdisc, Daegu, Korea). Three biological (10,000 ppm each) and three analytical replicates for each of the extracted samples were analyzed using both GC-TOF-MS and UHPLC-LTQ-IT-MS/MS methods. The samples for bioactivities assay were extracted using the similar procedure.

### 4.4. GC-TOF-MS Analysis

An Agilent 7890 GC system (Agilent Technologies, Palo Alto, CA, USA), equipped with an Agilent 7693 autosampler, was attached to a TOF Pegasus III mass spectrometer (Leco, St. Joseph, MI, USA), which was operated in electron ionization (EI) mode (70 eV). The column was an Rtx-SMS column (30 m length × 0.25 mm i.d. × 0.25 μm film thickness; Restek Corp., Bellefonte, PA, USA). Helium was used as the carrier gas, and was maintained at a constant flow of 1.5 mL/min. Then, 1 μL of the derivatized sample was injected in a split mode (10:1). The oven temperature was maintained at 75 °C for 2 min, increased to 300 °C at a rate of 15 °C/min, and then held at 300 °C for 3 min. The acquisition rate was set to 20 scans/s with a mass scan range of 45–1000 *m*/*z*. The injector and ion source temperatures were set at 250 °C and 230 °C, respectively.

### 4.5. UHPLC-LTQ-IT-MS/MS Analysis

The Thermo Fisher Scientific LTQ XL ion trap mass spectrometer consisted of an electrospray interface (Thermo Fisher Scientific, San Jose, CA, USA) coupled with a DIONEX UltiMate 3000 RS Pump, RS autosampler, RS column compartment, and RS diode array detector (Dionex Corporation, Sunnyvale, CA, USA). Each 10 μL sample was injected into and separated on a Thermo Scientific Syncronis C18 UHPLC column (100 mm × 2.1 mm i.d. × 1.7 μm particle size). The mobile phase consisted of water (A) and acetonitrile (B) with 0.1% formic acid (*v*/*v*) at a flow rate of 0.3 mL/min. The solvent gradient condition was increased from 10% to 100% of solvent B over 15 min, maintained for 3 min, and re-equilibrated to the initial condition for 4 min. The temperature of the column during measurement was maintained at 35 °C. The photodiode array was set at 200–600 nm for detection, and was managed by a three-dimensional (3D) field. The ion trap analysis was performed in full-scan ion modes within a range of 150–1000 *m/z*. The capillary temperature was tuned at 275 °C. The capillary and source voltages were set to 39 V and ±5 kV, respectively. Tandem MS analyses were performed using scan-type turbo data-dependent scanning (DDS) under the same conditions used for MS scanning.

### 4.6. UPLC-Q-TOF-MS Analysis

A Waters ACQUITY UPLC system (Waters Corp., Milford, MA, USA), equipped with a binary solvent delivery system, an autosampler, and a UV detector, was combined with a Waters Q-TOF Premier MS (Micromass MS Technologies, Manchester, UK) system. Aliquots (5 μL) of each sample were then injected into an ACQUITY BEH C18 column (100 mm × 2.1 mm i.d. × 1.7 μm particle size) at a flow rate of 0.3 mL/min. The mobile phase consisted of (A) water and (B) acetonitrile with 0.1% formic acid (*v*/*v*) at a flow rate of 0.3 mL/min. The gradient was linearly increased from 5% to 100% acetonitrile over 10 min, and was then decreased to 5% over 2 min. The mass spectrometer was operated in full-scan mass spectral range (100–1000 *m*/*z*), and the source temperature was 100 °C. The desolvation gas (nitrogen) and cone gas (nitrogen) flow rates were set at 700 L/h and 0.0 L/h at 300 °C, respectively. The capillary and cone voltages were set to 3.0 kV and 40 V, respectively.

### 4.7. Data Processing and Statistical Analysis

GC-TOF-MS raw data were converted to netCDF format (*.cdf) with ChromaTOF software (LECO). UHPLC-LTQ-ESI-IT-MS/MS raw data files were converted using the thermo file converter program in the Xcalibur™ Software (version 2.2, Thermo, San Jose, CA, USA). After conversion, the MS data were processed using the Metalign software package (http://www.metalign.nl) [60], and the resulting data were exported to an Excel (Microsoft, Redmond, WA, USA) file. Multivariate statistical analyses were processed using SIMCA-P+ (version 12.0, Umetrics, Umea, Sweden). We performed principal component analysis (PCA) and partial least-square discriminant analysis (PLS-DA) to compare metabolite differences between samples. Orthogonal projection to latent structures-discriminant analysis (OPLS-DA) was also performed to compare metabolite differences between the two samples: GB and UGB. Significantly different metabolites between the two samples were selected using VIP values > 0.7 and *p* < 0.05 as cutoffs. The *p*-values for different metabolite-based cluster groups were determined using STATISTICA (ver. 7.0; StatSoft, Tulsa, OK, USA).

### 4.8. Determination of Antioxidant Activities by ABTS and FRAP Assays

The ABTS assay was performed using the method of Re et al. with some modifications [61]. The stock solutions included 7 mM ABTS solution and 2.45 mM potassium persulfate solution. The working solution was prepared by mixing the two stock solutions in equal quantities and allowing them to react for 1 day at room temperature in the dark. The solution was then diluted until the absorbance reached 0.7 ± 0.02 at 734 nm using a spectrophotometer (Spectronic Genesys 6, Thermo Electron, Madison, WI, USA). Each ginseng extract (10,000 ppm) was reacted with 190 μL of the diluted ABTS solution for 7 min in the dark. The absorbance was then measured at 734 nm using a spectrophotometer. The standard curve was linear between 0.016 mM and 0.5 mM of Trolox equivalents. The results were expressed in mM Trolox equivalents per mg of dry weight of extract. For the FRAP assay [62], a mixture of 10 mM TPTZ solution in 40 mM HCl, 20 mM iron (III) chloride, and 300 mM acetate buffer at pH 3.6 (1:1:10, *v*/*v*/*v*) was used as the FRAP reagent. The analysis was performed by adding 300 μL of FRAP reagent to 10 μL of sample (2-fold dilution in 100% methanol) in a 96-well microplate, followed by a 6 min incubation at room temperature. The resulting absorbance was measured at 570 nm, and the results were expressed in mM of Trolox equivalent concentration/mg on a dry weight basis. Trolox (0.016–2 mM) served as a standard to quantify the antioxidant activities of the samples. All experiments were performed for 3-biological as well as analytical replicates of the extracted samples.

### 4.9. Determination of Total Phenolic and Flavonoid Contents

TPC in ginseng samples was determined according to the Folin-Ciocalteu colorimetric method, as modified by Singleton et al. [63]. A total of 100 μL of 0.2 N Folin-Ciocalteu’s phenol reagent was added to 20 μL of each sample in 96-well plates, followed by incubation in the dark for 5 min. Then, 80 μL of 7.5% sodium carbonate solution was added to the mixture and measured at 750 nm using a microplate reader (Spectronic Genesys 6). TPC was calculated on the basis of a standard curve with a gallic acid equivalent concentration (ppm). The standard solution concentration curve ranged from 7.813 to 500 ppm. All experiments were conducted in triplicate. TFC was measured, and 180 μL of 90% diethylene glycol, 20 μL of 1 N NaOH, and 20 μL of each sample extract were then mixed and incubated for 60 min at room temperature in the dark. Absorbance was measured at 405 nm using a microplate reader. The results were presented as naringin equivalent concentrations (ppm). The standard solution concentration curve ranged from 3.125 to 2000 ppm. All experiments were performed in triplicate, with similar ginseng extracts as those used for MS analysis.

## 5. Conclusions

Our study evaluated the effects of ultra-high pressure (UHP) treatments on the composite metabolomes for the three classes of ginseng products (white ginseng, red ginseng, and ginseng berries). We further discriminated the various ginseng samples based on their primary and secondary metabolites profiles. In particular, UHP-treated ginseng berry samples were shown to contain significantly discriminant metabolites with comparatively higher bioactivities. Hence, in agreement with the earlier reports, we may assume that the UHP treatment directly effects an increase in the levels of extractable primary and secondary metabolites in ginseng samples. Therefore, UHP treatment can be accredited to have widespread applications in the agro-food and post-harvest food processing industries.

## Figures and Tables

**Table 1:** Discriminant metabolites from the non-treated (GB) and UHP-treated (UGB) ginseng berry samples identified using GC-TOF-MS.

RT (min)	Metabolites ^a^	Mass	MS Fragment Ion ^b^	VIP	TMS	Ref ^c^
Amino Acids						
7.96	Serine	218	59, 100, 188, 204, 218	1.25	3	STD
8.21	Threonine	291	89, 101, 129, 203, 291	1.24	3	STD
9.35	Aspartic acid	232	100, 133, 188, 202, 232	1.06	3	STD
9.43	GABA	304	86, 100, 133, 216, 304	1.26	3	STD
9.78	Hydroxyglutaric acid	203	85, 129, 157, 203, 247	1.28	3	MS
10.13	Glutamic acid	246	84, 100, 204, 203, 246	1.28	3	STD
10.56	Asparagine	231	100, 116, 132, 188, 231	1.24	3	STD
14.26	Tryptophan	202	100, 131, 202, 218, 291	1.23	3	STD
Organic Acids						
4.93	Lactic acid	191	88, 101, 133, 191, 219	1.27	2	STD
5.74	Ethylmalonic acid	189	61, 103, 161, 189, 204	0.82	1	MS
9.08	Malic acid	233	101, 133, 189, 233, 335	1.28	3	STD
10.19	Benzoic acid	267	91, 193, 223, 267, 282	1.27	2	MS
11.54	Shikimic acid	255	93, 167, 189, 204, 255	1.28	4	STD
11.66	Citric acid	273	133, 245, 273, 319, 465	1.28	4	MS
12.96	Gluconic acid	171	89, 103, 129, 189, 217	1.25	5	STD
Sugars and Sugar Derivatives						
7.69	Glyceric acid	292	59, 89, 133, 189, 292	1.17	3	MS
9.27	*meso*-Erythritol	217	103, 133, 189, 205, 307	1.27	4	MS
11.01	Ribitol	319	103, 129, 189, 217, 319	1.14	5	STD
12.26	Galactose	319	103, 129, 160, 189, 205	1.23	5	STD
12.39	Glucose	319	103, 129, 189, 205, 319	1.28	5	STD
13.52	*myo*-Inositol	265	103, 129, 191, 265, 305	1.27	6	STD
16.58	Turanose	361	103, 129, 169, 191, 361	1.10	7	MS
Fatty Acids						
14.06	Linoleic acid	262	55, 81, 129, 164, 262	1.24	1	STD
14.22	Stearic acid	341	95, 129, 159, 195, 341	1.19	1	STD

^a^ Metabolites identified based on the variable importance projection (VIP) analysis results (with a cut-off value of 0.7) and *p* < 0.05; ^b^ MS fragmentation is the fragmentation of the tentative compound; ^c^ MS mass spectrum was consistent with those of NIST and in-house libraries. Standard compound (STD) mass spectrum was consistent with that of the standard compounds. Retention time (RT), trimethylsilyl (TMS), Identification (ID).

**Table 2:** Discriminant metabolites from the non-treated (GB) and UHP-treated (UGB) ginseng berry samples identified using UHPLC-LTQ-IT-MS/MS.

RT (min)	Tentative Metabolite ^a^	UHPLC-LTQ-IT-MS/MS				UPLC-Q-TOF-MS			ID ^d^
		[M − H]^−^	[M + H]^+^	MS^n^ Fragment Ions ^b^	UV λmax (nm)	Measured [M − H]^−^	M. F.	Error (mDa) ^c^	
7.05	N.I. (1)	563	565	563 > 282 > 265	296, 321, 504	-	_-_	-	-
8.01	N.I. (2)	1174	1176	-	264, 317	-	_-_	-	-
8.76	Notoginsenoside R1	977	933	977 > 931	277	931.5114	C_43_H_80_O_21_	0.2	STD
9.18	Ginsenoside-Re	991	947	991 > 945 > 779	278	945.5423	C_48_H_80_O_18_	0.9	STD
9.56	Malonyl-ginsenoside Re	1031	1033	1031 > 987 > 945	281	1031.5427	C_51_H_84_O_21_	0.1	[25,26]
10.48	Ginsenoside Rb1	1153	1109	1153 > 1107	271	1107.5951	C_54_H_84_O_23_	0.1	STD
10.75	Notoginsenoside R2	815	771	769 > 637	264	769.4738	C_41_H_70_O_13_	−1.8	[25,26]
11.29	Ginsenoside Rd	991	947	991 > 946	267	945.5423	C_48_H_80_O_18_	0.5	STD
11.40	Malonylginsenoside Rd	1031	1033	945 > 783 > 621	267, 366	1031.5423	C_51_H_84_O_21_	−0.8	[26]
11.60	Malonylginsenoside Rd/isomer	1031	1033	945 > 783 > 621	281	1031.5427	C_51_H_84_O_21_	0.8	[26,27]
11.89	Notoginsenoside Rt1	961	917	961 > 915 > 783	215	915.5315	C_47_H_80_O_17_	0.8	[25]
12.05	Notoginsenoside Rt1/isomer	961	917	961 > 915 > 783	215	915.5317	C_47_H_80_O_17_	0.6	[25]
12.38	Malonylnotoginsenoside Rt1	1001	1003	-	216	-	-	-	[25]
12.79	Ginsenoside Rg3	829	785	829 > 783,621	217	783.4836	C_42_H_72_O_13_	-4.0	STD
13.70	N.I. (3)	869	871	-	218	-	-	-	-

^a^ Metabolites identified based on the VIP analysis results (with a cut-off value of 0.7) and *p* < 0.05; ^b^ MS^n^ fragment patterns detected in negative ion mode; ^c^ Differences between observed mass and calculated mass; Error in milliDalton (mDa); Molecular formula (M.F.); ^d^ Identification: Standard compound (STD); References (Ref.); Retention time (RT); Not identified (N.I.).

## Supplementary Materials

Supplementary materials can be accessed at: http://www.mdpi.com/1420-3049/21/6/816/s1.
