# Supplementary material for: Ultrahigh Pressure Processing Produces Alterations in the Metabolite Profiles of Panax ginseng
Source: Molecules. 2016 Jun 22;21(6):816. doi: 10.3390/molecules21060816 (PMC6273588; doi:10.3390/molecules21060816)
Supplement: Supplementary file 1 [file molecules-21-00816-s001.pdf]

Supplementary Materials: Ultrahigh Pressure Processing Induces Alterations in the Metabolite Profiles of *Panax ginseng*

Mee Youn Lee, Digar Singh, Sung Han Kim, Sang Jun Lee and Choong Hwan Lee

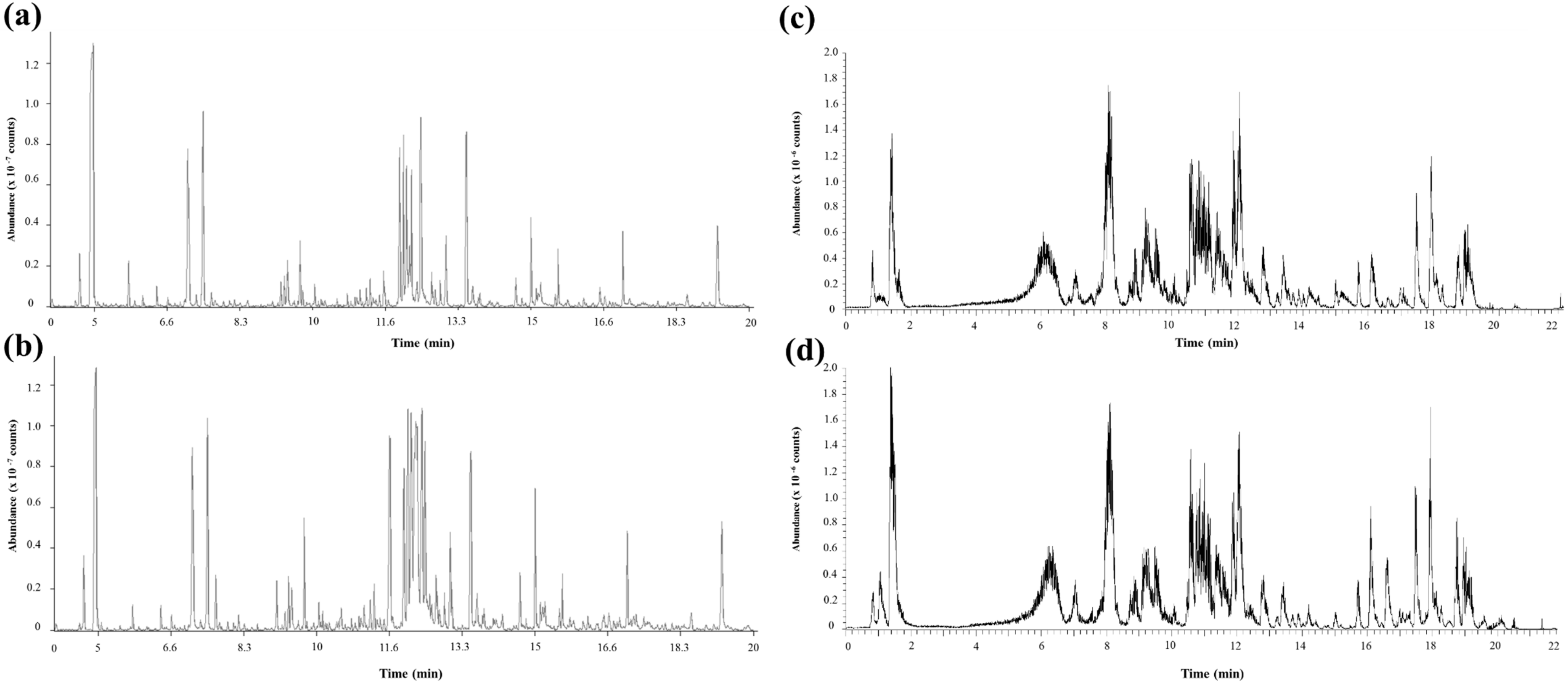

Figure S1. Chromatogram of the non-treated ginseng berry (GB) (**a**,**c**) and the UHP-treated ginseng berry (UGB) (**b**,**d**) extract analyzed with GC-TOF-MS (**a**,**b**) and UHPLC-LTQ-MS/MS in positive mode (**c**,**d**).

Table S1. The average relative levels of the significantly discriminant metabolites in GB and UGB extracts indicated in the respective steps of the metabolic pathway shown in Figure 4.

Compound Name	Average of Peak Area [a]					
	GB			UGB		
Primary Metabolites						
Amino Acids						
Serine	8252	±	1542	24,504	±	1502
Threonine	2460	±	316	6288	±	482
Aspartic acid	147,876	±	25,276	256,899	±	4995
GABA	58,386	±	3424	43,316	±	612
Hydroxyglutaric acid	21,783	±	847	9051	±	140
Glutamic acid	18,332	±	2996	81,938	±	1661
Asparagine	8529	±	3120	33,419	±	1477
Tryptophan	54,087	±	20,594	192,891	±	9137
Organic acids						
Lactic acid	352,677	±	6851	338,098	±	7584
Malic acid	4691	±	757	57265	±	1102
Benzoic acid	5227	±	310	6318	±	197
Shikimic acid	3911	±	281	4308	±	93
Citric acid	35,249	±	1258	614,765	±	12,055
Gluconic acid	92	±	42	431	±	12
Sugars and Sugar Derivatives						
Glyceric acid	9823	±	9261	53,848	±	7488
meso-Erythritol	78,280	±	3644	52,156	±	904
Ribitol	2378	±	168	1287	±	674
Glactose	204,056	±	3946	236,850	±	3790
Glucose	31,282	±	1911	500,324	±	9188
myo-Inositol	150,474	±	4444	136,641	±	3691
Turanose	32,177	±	8572	17,253	±	1971
Ethylmalonic acid	2499	±	187	2837	±	286
Fatty Acids						
Linoleic acid	975	±	62	840	±	35
Stearic acid	2693	±	69	3085	±	94
Secondary Metabolites						
Notoginsenoside R1	3,041,114	±	78,123	4,038,299	±	152,690
Ginsenoside-Re	2,519,807	±	107,869	3,726,045	±	175,049
Ma-Ginsenoside Re	4911398	±	241,612	6,497,414	±	300,877
Ginsenoside-Rb1	1048915	±	46,579	1,471,512	±	66,716
Notoginsenoside R2	6906175	±	333,683	8,793,301	±	409,718
Ginsenoside-Rd	278,342	±	22,995	366,493	±	37,867
Ma-Ginsenoside Rd	988233	±	94,161	1,308,145	±	65,951
Notoginsenoside Rt1	6138233	±	351,518	6,964,508	±	252,809
Ma-Notoginsenoside Rt1	345,271	±	23,170	384,138	±	26,874
Ginsenoside-Rg3	950,600	±	54,404	1,133,790	±	45,576

[a] Data are presented as mean ± SD.
